# Adaptive Development of Soil Bacterial Communities to Ecological Processes Caused by Mining Activities in the Loess Plateau, China

**DOI:** 10.3390/microorganisms8040477

**Published:** 2020-03-27

**Authors:** Zhanbin Luo, Jing Ma, Fu Chen, Xiaoxiao Li, Qi Zhang, Yongjun Yang

**Affiliations:** 1 School of Environment Science and Spatial Informatics, China University of Mining and Technology, Xuzhou 221008, China; lzbin1991@cumt.edu.cn (Z.L.); lixiaoxiao@cumt.edu.cn (X.L.); zhangqi2019@cumt.edu.cn (Q.Z.); y.yang@cumt.edu.cn (Y.Y.); 2 College of Resources and Environment, Northwest A&F University, Yangling 712100, China; 3 Low Carbon Energy Institute, China University of Mining and Technology, Xuzhou 221008, China; jingma2013@cumt.edu.cn

**Keywords:** soil bacteria, molecular ecological network, mining subsidence, vegetation rehabilitation, Loess Plateau

## Abstract

Microorganisms are the driving force behind the circulation and transformation of the soil substance. The development of soil bacterial communities is critical for ecosystem restoration and evolution. In the Loess Plateau, coal mining activities have aggravated the deterioration of the fragile local ecological environment. The adaptive development of soil bacterial communities in response to different ecological processes caused by coal mining activities was explored through high-throughput sequencing technology and an ecological network analysis of the mining subsidence area of the Daliuta Coal Mine and vegetation rehabilitation area of the Heidaigou Coal Mine in the Loess Plateau. The results showed that while mining subsidence was inhibited, vegetation rehabilitation promoted the soil physicochemical properties. Soil organic matter, available phosphorus and available potassium in the subsidence area decreased significantly (*P < 0.05*), while soil organic matter, soil water, pH and EC in the vegetation rehabilitation area increased significantly (*P < 0.05*). The diversity index in the subsidence area decreased by about 20%, while that in the vegetation rehabilitation area increased by 63%. Mining subsidence and vegetation rehabilitation had a distinct influence on the molecular ecological networks of the soil bacteria, which tended to be more complex after the mining subsidence, and the number of connections in the network increased otherwise significantly enhanced interactive relationships. After the vegetation rehabilitation, the number of modules in the ecological network increased, but the contents of modules tended to be simpler. Soil bacterial communities adapted to the changes by changing the relationships between bacteria in response to different ecological processes. This study provides new insights into the monitoring and abatement of the damaged ecological environment in mines.

## 1. Introduction

Coal has been an important propellant for industrial development [1]. Currently, China is the highest producer and consumer of coal in the world [2,3]. The Loess Plateau is the most important coal production base, constituting half of the coal production capacity of China. However, the ecological environment in this region is fragile, with serious land degradation, and the urgent need for ecological restoration [4,5]. While coal mining promotes the local economy, it also seriously damages the local natural environment [6,7]. With the increasing intensity of conflict between mining and ecological protection, the sustainable development of local society and the economy is hindered. This conflict has attracted widespread attention [8,9,10].

Coal mining interferes with ecosystems in several ways [8]. From 1987 to 2014, 1.3651 million hectares of land in China was damaged due to coal mining, with an average annual loss of 48,800 hectares [11]. The local ecological process changed due to surface excavation, crack, collapse, deep plowing, and waste stacking, resulting in various changes in soil physicochemical properties, seed banks and the microbial population [4,7,8,12,13,14]. Mining subsidence is an important phenomenon associated with underground mining, which causes the stretching, squeezing and fracture of soil, resulting in the greater change of soil moisture [15,16], decrease of soil bulk density [17,18], a decrease of total soil porosity [19], and enhanced soil desertification [20]. Moreover, mining subsidence also changes the distribution and transport of soil nutrients, resulting in the decrease of pH [21], loss of organic matter [22], and the decrease of nitrogen, phosphorus and potassium contents [23]. Vegetation rehabilitation is an important remediation project for the ecological restoration of damaged mines, which can effectively improve the soil quality [24,25], improve the efficiency of soil water uptake [26], accelerate the accumulation of soil organic carbon [22,27], and protect biodiversity [28]. However, the influence of mining activities in different areas is usually complex, and normal principles do not quite apply. Therefore, the influence of coal mining activities on ecosystems should be discussed based on practical scenarios.

Microorganisms are the most active components in the soil environment and play key roles in organic matter decomposition and the cycling of nutrients [29,30,31,32]. Soil microorganisms are also very sensitive to external changes, so that they can serve as the indices to land degradation and ecological restoration [33,34]. With the development of sequencing technology, revealing the relationships between bacterial community structures and functions has become a highlight in ecological research [35,36]. Over recent years, soil bacterial communities in coal mining areas have drawn attention. Mining subsidence changes the support of the soil environment, and thereby negatively affecting the adaptability of soil bacteria and a decline in the number and activity of bacteria [37,38]. Even after 20 years of land reclamation in the coal mining area, the number of soil bacteria was still lower than that in an undisturbed area, but shrub coverage played a key role in ecological restoration [39]. Furthermore, vegetation rehabilitation could not only improve the richness of soil bacteria, but also promote the bacterial metabolism in original soil [29], which was found to be much higher than that in uncultivated soil [40,41]. After 5-14 years of vegetation rehabilitation, significant interactions were observed between plants and bacteria [42]. Some scholars suggest that the structure of soil bacteria after vegetation rehabilitation was dependent on pH and other abiotic characteristics [30]. Soil bacteria could serve as sensitive indicators of land degradation and ecological restoration [43,44]. However, previous studies on the impact of mining subsidence and vegetation rehabilitation on soil bacteria focused mainly on the changes in soil bacterial community structures, and not on the internal interactive relationships between bacterial communities. The development of bacterial communities and crucial bacterial microbes are still unclear.

Molecular ecological network analysis, as a recently developed method, can visually describe the complex potential structures and interactions among bacterial communities. This method is innovative in revealing the stability and complexity of ecological processes and functions [45,46,47,48,49]. Zhou et al. [50] and Lu et al. [51] studied the relationships and differences of soil bacterial communities in network structures, interactions and crucial species, under the conditions of high CO_2_ levels and continuous cropping of potato. Another study reported that increased precipitation enhanced the connections between the molecular ecological networks of soil bacteria [52]. As an intense surface disturbance, coal mining impacts the soil bacterial community [53]. Recently, we also observed that cracks reinforced the interactions among soil bacterial communities in the coal mining area of Loess Plateau [14]. However, due to technical limitations, the influence of mining subsidence and vegetation rehabilitation on the interactions between soil bacterial species has not been extensively evaluated.

Therefore, here, the mining subsidence area of the Daliuta Coal Mine and the vegetation rehabilitation area of the Heidaigou Coal Mine in the Loess Plateau were studied to disclose: (1) the influence of mining subsidence and vegetation rehabilitation on the soil bacterial community structure; (2) the changes in the soil bacterial molecular ecological network after the mining subsidence and vegetation rehabilitation; (3) the interactions between soil bacterial communities and those between environmental factors caused by mining subsidence and vegetation rehabilitation. This study would also be theoretically beneficial for the ecological restoration of damaged mining areas in the Loess Plateau and shed light on the construction of green mines.

## 2. Materials and Methods

### 2.1. Site Description

**Figure 1:**
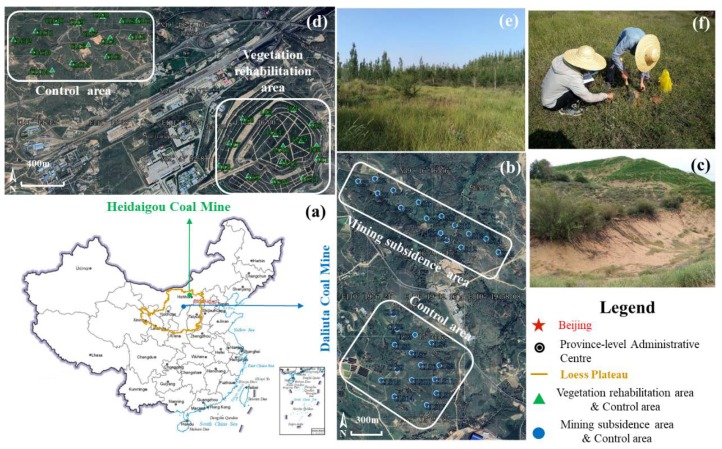
The study area and sampling sites of coal mining substance and vegetation rehabilitation. (**a**) Location of Daliuta and Heidaigou Coal Mines; (**b**) Sampling points of mining subsidence and control area in Daliuta Coal Mine; (**c**) Photograph of mining subsidence in Daliuta Coal Mine; (**d**) Sampling points of vegetation rehabilitation and control area in Heidaigou Coal Mine; (**e**) Photograph of vegetation rehabilitation in Heidaigou Coal Mine; (**f**) Photograph of soil sampling.

The Loess Plateau is rich in coal resources, with a long history of coal mining, but the ecosystem has been greatly damaged (Figure 1a). In this study, the mining subsidence area of the Daliuta Mine in the northern part of the Loess Plateau and vegetation rehabilitation area of the Heidaigou Mine in Inner Mongolia Province were selected as the target regions. The locations of both regions are adjacent, and their climates, soil types, and soil textures are similar.

The Daliuta Coal Mine is located at 110°12′ E‒110°23′ E and 39°13′ N‒39°22′ N, with a warm semi-arid continental climate and obvious seasonal changes (Figure 1b). The altitude is 1057‒1334 m. The average annual temperature is 8.6 °C, and annual precipitation is 290.4‒410.3 mm. The Daliuta Coal Mine was officially established in the year of 1996, with an original production capacity of 3.6 million tons/year, which has increased to 10.4 million tons/year currently. The underground mine goaf accounts for 70% of the total mining area. Long-term mining has led to a decline in the groundwater level, thickening of the surface dry sand layer, decrease in the soil moisture content, and frequent loss of vegetation, as shown in Figure 1c, and is a typical mining subsidence area in the Loess Plateau. The sampling site in this study was located in the southeast part of the Daliuta Mine. It belonged to the 52302 working face, a surface at which underground coal mining activities were carried out. The coal seam was shallowly buried, thick, and almost horizontal. The surface was covered by a thick, loose layer of soil. Long-arm mining and caving roof management were adopted with an advancing speed of 12 m·d^−1^. The mining pattern here was a typical high-strength mining mode with a super-large working face.

The Heidaigou Mine is located at 39°43′‒39°49′ N and 111°13′‒111°20′ E, with a warm semi-arid continental climate and obvious seasonal changes (Figure 1d). The annual precipitation is 231.0‒459.5 mm, the annual average temperature is 7.2 °C, and the annual average evaporation is 2082.2 mm, with an annual 3119.3 sunshine-hours. The typical vegetation of the Heidaigou Coal Mine is savanna in the temperate zone. The plants are low and scarce, with less than 30% coverage. The soil is slightly alkaline, infertile and aeolian sandy. The Heidaigou Coal Mine was operated in 1996, leading to serious land damage. The sampling sites were located in the northern fields with 16 years of vegetation rehabilitation and remote fields with no vegetation rehabilitation (Figure 1e).

### 2.2. Soil Sample Acquirement and Analysis

Fifteen samples for testing and 15 control samples were collected from each of the two mining regions between August 15 to 23, 2018 (Figure 1f). A total of 60 samples were collected. All sampling sites were selected with similar vegetation types, which were mainly covered by *Stipa bungeana*. The surface soil in the subsidence area of the Daliuta Mine was denoted by LS, and the control samples were denoted by CLS (Figure 1b). The surface soil in the vegetation rehabilitation area of the Heidaigou Mine was denoted by LR, and the control samples, acquired from land far away from the vegetation rehabilitation area, were denoted by CLR (Figure 1d). During the sampling process, about 1 kg of topsoil was randomly withdrawn at a depth of 0-20 cm using the five-point sampling method. These soil samples were packed and sealed in sterile polyethylene bags, frozen at -20 °C in a refrigerator and sent to the laboratory for treatment. A proportion of fresh soil samples were directly analyzed to learn the structure of soil bacterial communities. The other samples were naturally dried. Then, the gravels and residues from organisms were removed. The physicochemical properties of these samples were determined after screening with 2-mm sieves.

### 2.3. Analysis of Soil Physicochemical Properties

The moisture content, temperature, pH, EC, organic matter, ammonium nitrogen, nitrate nitrogen, available potassium and available phosphorus of soil samples were measured through the procedures mentioned henceforth [54]. The moisture content and temperature were measured using a fast determination instrument (TR-6, Shunkeda, Guangzhou, Guangdong Province, China). The pH and EC were measured by potentiometry (water: soil = 1: 2.5; DDS-307A, Leici Company, Shanghai, China). The organic matter content was determined through the potassium dichromate-colorimetry (Specord 210Plus, Jena Company, Jena, Germany). The ammonium nitrogen content was analyzed by ultraviolet spectrophotometry after potassium chloride extraction. The nitrate-nitrogen content was analyzed by ultraviolet spectrophotometry after calcium chloride extraction. The available phosphorus content was analyzed by molybdenum-antimony-scandium colorimetry after ammonium bicarbonate. Available potassium was determined by flame photometry after ammonium acetate extraction (FP640, Jingke, Shanghai, China).

### 2.4. DNA Extraction, Purification, PCR Amplification, and 16SrRNA Sequencing

DNA from 60 soil samples was extracted according to the instructions of FastDNA™ SPIN kit (MP Biomedicals, Irvine, CA, USA), and the V4 and V5 regions of corresponding 16S rRNA in soil bacteria were PCR amplified using primers 515F(5′-GTGCCAGCMGCCGCGGTAA-3′) and 907R(5′-CCGTCAATTCMTTTRAGTTT-3′). The PCR amplification procedure was a follows: pre-denaturation at 98 °C (2 min); 25 cycles of denaturation at 98 °C (15 s), annealing at 55 °C (30 s), extension at 72 °C (30 s); extension at 72 °C (5 min), and cooling to 10 °C. The products of PCR amplification were detected by 2% agarose gel electrophoresis. The target sequences were recovered with a gel recycling kit (Axygen, Corning, NY, USA). According to the preliminary electrophoresis quantitation results, the sequences recovered were quantified on a Microplate reader (BioTek, FLx800, Winooski, VT, USA), using Quant-iT PicoGreen dsDNA Assay Kit fluorescent reagent. The samples were mixed in equal proportion. The sequencing library of soil bacteria was prepared with the TruSeq Nano DNA LT Library Prep Kit (Illumina, San Diego, CA, USA). The constructed library was quantified by Qubit and Q-PCR and then sequenced (Shanghai Personal Biotechnology Co., Ltd., Shanghai, China) with HiSeq2500 PE2500 (Illumina, San Diego, CA, USA).

### 2.5. Construction of Soil Bacterial Molecular Ecological Network

The molecular ecological network of soil bacteria was constructed with 16S rRNA sequencing data, according to the theory of random matrix (RMT) [46,50,52]. Four networks were built with the 60 acquired samples, and 15 samples were involved in each network, which contained corresponding soil types. During the construction process, the OTUs (operational taxonomic units) with frequencies lower than 75% in the samples were abandoned, and the relative abundance values of the logarithms of OTU data (log10) were transformed to calculate the Pearson correlation coefficients between OTUs to acquire similarity matrixes. With optimal similarity threshold values, an adjacency matrix was generated from the similarity matrix. The strength of the connection between each pair of nodes was coded with the adjacency matrix. The ecological community was predicted by analyzing the nearest distance distribution of the eigenvalues of the correlation matrix. The network construction and statistical analysis were performed by using the platform http://ieg4.rccc.ou.edu/mena. The molecular ecological network of soil bacteria after mining was visualized by the software Cytoscape 3.7.1 [55].

### 2.6. Statistical Analysis and Processing

The richness and diversity of soil bacterial communities were analyzed using Alpha diversity indexes at the galaxy platform (http//:mem.rcees.ac.cn.8080/). This was carried out by using the Chao1 estimator (https://mothur.org/wiki/chao/) and ACE estimator (https://mothur.org/wiki/ace/), reflecting the soil bacterial community richness, as well as using the Shannon diversity index (https://mothur.org/wiki/shannon/) and the Simpson index (https://mothur.org/wiki/simpson/), representing the soil bacterial community diversity. The one-way analysis of variance (ANOVA) was carried out with the SPSS 20.0 software (IBM, Armonk, NY, USA). A redundancy analysis (RCA) was applied to characterize the interactions between soil environmental factors and bacterial communities with Canoco 4.5 for windows software. Mantel Test analysis was conducted with the R-project software (https://www.r-project.org/). The abundance plots of bacterial communities at different levels were obtained with the Origin 9.0 software (Origin Lab, Northampton, MA, USA).

## 3. Results

### 3.1. Effects of Ecological Processes on the Soil Physicochemical Characteristics in the Mining Subsidence and Vegetation Rehabilitation Areas

The ecological processes dominated by mining subsidence and vegetation rehabilitation exhibited remarkable differentiations on the soil physicochemical properties (Table 1). While the mining subsidence was inhibited, vegetation rehabilitation recovered the soil physicochemical properties after disturbance due to coal mining.

The mining subsidence had a negative impact on soil physicochemical properties. In the mining subsidence area, soil temperature (ST), organic matter (OM), available phosphorus (AP), available potassium (AK), ammonium nitrogen (AN), and nitrate nitrogen (NN) decreased by 5.1 °C (*P < 0.01*), 42.2% (*P < 0.01*), 20 mg·kg^−1^ (*P < 0.05*), 20 mg·kg^−1^ (*P < 0.05*), 0.2 mg·kg^−1^ (*P < 0.05*), and 0.2 mg·kg^−1^ (*P > 0.05*), respectively. Concomitantly, soil water content (SWC), pH, and electrical conductivity (EC) increased by nearly 33.1% (*P < 0.05*), 0.2 (*P > 0.05*), and 0.3 ms·cm^−3^ (*P > 0.05*), respectively. Mining subsidence had caused a decreasing tendency of ST, OM, AP, AK, AN, and NN, but an increasing trend of SWC, pH, and EC.

The vegetation rehabilitation exhibited positive influence on soil physicochemical properties. In the vegetation rehabilitation area, SWC, pH, EC, OM, AP, AK, NN, and AN increased by 25.1% (*P < 0.05*), 0.8 (*P < 0.001*), 3.5 ms·cm^−3^ (*P < 0.05*), 0.4 g·kg^−1^ (*P < 0.01*), 30 mg·kg^−1^ (*P < 0.01*), 20 mg·kg^−1^ (*P < 0.01*), 0.2 mg·kg^−1^ (*P < 0.001*), and 0.2 mg·kg^−1^ (*P < 0.05*), respectively. However, ST declined significantly by 5.2 °C (*P < 0.001*). Thus, vegetation rehabilitation initiated a recovery trend of SWC, pH, EC, OM, AP, AK, NN, and AN, but a decline in ST.

### 3.2. Effects of Ecological Processes on the Soil Bacterial Communities in Mining Subsidence and Vegetation Rehabilitation Areas

#### 3.2.1. Effects of Mining Subsidence and Vegetation Rehabilitation on the Alpha Diversities of Soil Bacteria

**Figure 2:**
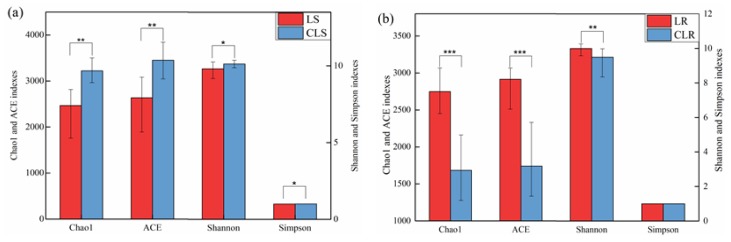
Changes in soil bacterial alpha diversities in mining subsidence area and vegetation rehabilitation area. (**a**,**b**) are the index of alpha diversity in Daliuta Coal Mine and Heidaigou Coal Mine, respectively. LS and CLS represent samples from the mining subsidence area and control area in Daliuta Coal Mine, respectively. LR and CLR represent samples from vegetation rehabilitation area and control area in Heidaigou Coal Mine, respectively. * *p* < 0.5, ** *p* < 0.01, *** *p* < 0.001

The impact of mining subsidence and vegetation rehabilitation on the soil bacterial alpha diversities has been shown in Figure 2. The results show that the average Chao1 and ACE indices in the mining subsidence area of the Daliuta Coal Mine largely decreased by 20% (*P < 0.01*) compared to those in the control group. The Shannon and Simpson indices were slightly lower than those in the control group (*P < 0.05*). These characteristics indicate that the mining subsidence reduced the abundance and homogeneity of soil bacterial communities. The Chao1 and ACE indices in the vegetation rehabilitation area of the Heidaigou Coal Mine were 1.63 and 1.67 times of those in the control group, respectively, with significant differences (*P < 0.001*). These features indicate that the vegetation rehabilitation led to a significant increase in the diversity of the soil bacterial community structure.

#### 3.2.2. Effects of Mining Subsidence and Vegetation Rehabilitation on the Soil Bacterial Composition

**Figure 3:**
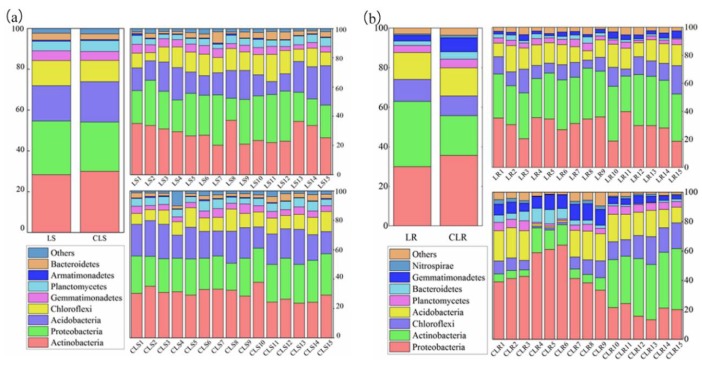
Bar diagram to represent the analysis at phylum level in mining subsidence area (**a**) and vegetation rehabilitation area (**b**).

The ecological processes dominated by mining subsidence and vegetation rehabilitation altered the compositions of soil bacterial communities (Figure 3). As shown in Figure 3a, the primary phyla in the soil sample of the mining subsidence area and control area of the Daliuta Coal Mine were consistent. The primary phyla, whose relative abundance accounted for more than 90% of the overall abundance, included *Actinobacteria*, *Proteobacteria*, *Acidobacteria*, *Chloroflexi*, *Gemmatimonadetes*, *Planctomycetes*, *Armatimonadetes*, and *Bacteroidetes*. However, the relative abundance of individual phylum appeared to have obviously changed. The relative abundance values of *Proteobacteria*, *Chloroflexi*, *Gemmatimonadetes*, and *Bacteroidetes* were elevated in the mining subsidence area, particularly, that of *Chloroflexi*, which increased to 25.18% (21.86% in the control group). The relative abundance of *Actinobacteria*, *Acidobacteria*, and *Planctomycetes* decreased slightly, particularly that of *Planctomycetes*, which decreased to 4.45% (7.97% in the control group). These changes indicate that the soil bacterial communities adapted according to the mining subsidence environment.

The primary phyla in the soil sample of the vegetation rehabilitation area and control area of the Heidaigou Coal Mine were similar (Figure 3b). The primary phyla, whose relative abundance accounted for greater than 90%, included *Proteobacteria*, *Actinobacteria*, *Chloroflexi*, *Acidobacteria*, *Planctomycetes*, *Bacteroidetes*, *Gemmatimonadetes*, and *Nitrospira*. Among them, the *Actinobacteria* preferred the soil environment in the vegetation rehabilitation area, of which the relative abundance was higher than that in the control group by 64.0%. However, the relative abundance values of *Proteobacteria* and *Gemmatimonadetes* were lower than those in the control group. The relative abundance of *Nitrospira* was even as low as < 1%. These changes demonstrated that the soil bacterial communities adapted to the vegetation rehabilitation environment.

### 3.3. Effect of Ecological Process on the Soil Bacterial Molecular Ecological Network in Mining Subsidence Area and Vegetation Rehabilitation Area

#### 3.3.1. Effect of Mining Subsidence and Vegetation Rehabilitation on Topological Properties of Soil Bacterial Molecular Ecological Networks

The specific topological properties of soil bacterial molecular ecological networks in mining subsidence and vegetation rehabilitation are mentioned in Table 2. According to the construction of molecular ecological networks, the OTUs with frequencies higher than 70% were adopted as the input items. The similarity thresholds for mining subsidence and vegetation rehabilitation areas were set to be 0.81 and 0.84, respectively. The average connectivity, average path length, average aggregation coefficient and modularity values of both constructed networks were greater than those of random networks, indicating that these networks were reliable. The number of connection lines in the subsidence area was greater than that in the control group, indicating the enhanced complexity in the molecular ecological network after mining subsidence. The average clustering coefficient, average path distance and density in the subsidence area were higher than those in the control group, indicating that the increase in the connection strength of each node and the network nodes were closer after the subsidence. However, the modularity index was smaller than that of the control group after the subsidence, indicating that the resistance of soil bacterial communities to external changes was weakened by subsidence. Compared to the control group, the number of nodes in the molecular ecological network of the vegetation rehabilitation area increased, although, the number of connection lines decreased, indicating that the community structure tended to be diversified, even when the interconnections were weakened.

**Figure 4:**
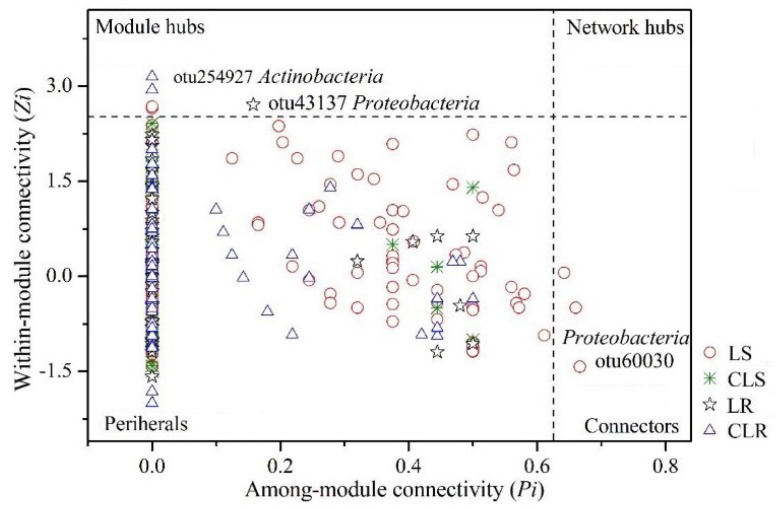
*Zi–Pi* plot of molecular ecological networks in mining subsidence area and vegetation rehabilitation area.

The modules and connectivity of molecular ecological networks in the mining subsidence area and vegetation rehabilitation area are shown in Figure 4. The connectivity within or between modules of a molecular ecological network can reflect the roles played by different OTUs. The nodes with *Zi* ≥ 2.5 or *Pi* ≥ 0.62 are generally considered as crucial species [46]. The ecological network of the subsidence area contained three connection nodes and one module, while that of the control group had only peripheral nodes. The ecological network of the vegetation rehabilitation area contained only one module, while that of the control group contained two modules. Among the key species, the nodes with the highest connectivity were located above the dotted line and towards the right side. The key species with the highest connectivity in the mining subsidence area is *Proteobacteria* while those in the vegetation rehabilitation area and the control group are *Proteobacteria* and *Actinobacteria*, respectively.

#### 3.3.2. Assessment of the Effect of Mining Subsidence and Vegetation Rehabilitation on Soil Bacterial Molecular Ecological Networks

**Figure 5:**
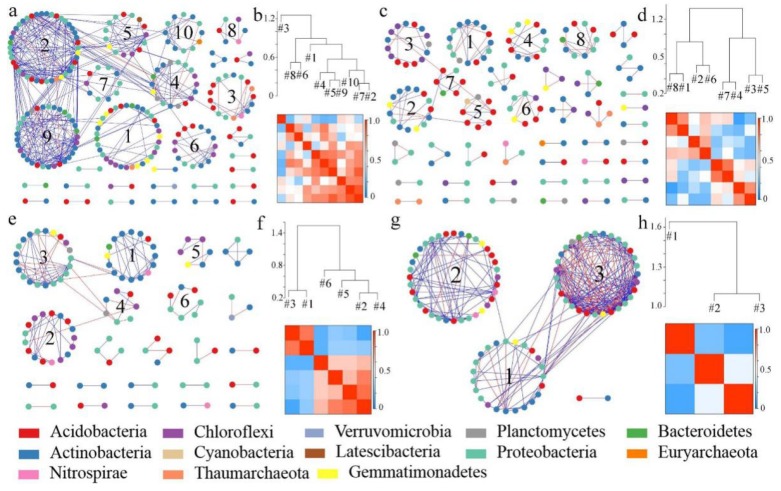
Soil bacterial molecular ecological networks and module eigengene hierarchy structure in a mining subsidence area and vegetation rehabilitation area. (**a**,**c**) represent the soil bacterial molecular ecological network in the mining subsidence area and its control. (**e**,**g**) represent the soil bacterial molecular ecological network in vegetation rehabilitation area and its control. (**b**,**d**) represent the soil bacterial modular eigengene hierarchy structure in mining subsidence area and its control. (**f**,**h**) represent soil bacterial modular eigengene hierarchy structure in the vegetation rehabilitation area and its control. The connecting line represents the interaction of the target level on phylum, with blue representing positive and red representing negative interaction.

Based on the sequencing results of soil bacteria, four visualized molecular ecological networks were constructed to show the relationships between species in both ecological processes (Figure 5). Figure 5a shows that after mining subsidence, significantly higher connections between the nodes were observed than those in the control group (Figure 5c) and that the nodes tended to cooperate with each other (blue). Figure 5b,d show the decline in the number of modules in the molecular ecological network of the subsidence area, although these modules exhibited closer connections, indicating that the soil bacteria made changes to adapt to the nutrient loss in the adverse habitat caused by subsidence and that these soil bacterial communities cooperated with each other. The connections between nodes in the network of vegetation rehabilitation areas were significantly lower than those in the network of the control group, as shown in Figure 5e,g, respectively. In addition, the number of network modules in the network of the vegetation rehabilitation area was greater than that in the control group. The relationships between modules appeared to change from competition (Figure 5h) to cooperation (Figure 5f). The network modularity index of the vegetation rehabilitation area was significantly higher than that of the control group, indicating that the resistance characteristics of bacteria to external changes were significantly enhanced, and that the vegetation rehabilitation had a positive impact on the bacterial communities.

### 3.4. Effects of Ecological Processes on Interactions between Soil Bacterial Communities and Physicochemical Properties in the Mining Subsidence and Vegetation Rehabilitation Areas

#### 3.4.1. Interactions between Soil Bacterial Phyla and Physicochemical Properties in the Mining Subsidence and Vegetation Rehabilitation Areas

**Figure 6:**
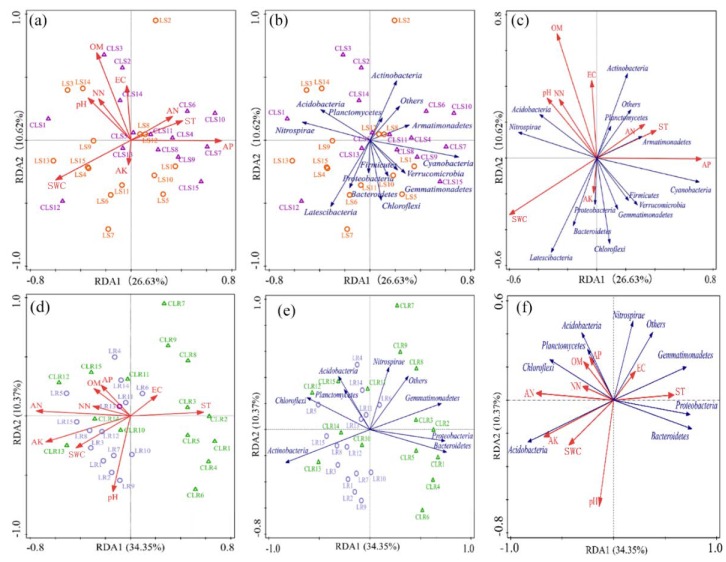
RDA analysis of samples and environmental factors (**a**,**d**), samples and major phyla (**b**,**e**), and environmental factors and major phyla (**c**,**f**). (**a**–**c**) represent RDA analysis of mining subsidence area and its control area in the Daliuta Coal Mine. (**d**–**f**) represent RDA analysis of vegetation rehabilitation area and its control area in the Heidaigou Coal Mine.

At the level of phylum > 1%, RDA analysis of soil bacterial communities and environmental factors was carried out with the subsidence area of the Daliuta Coal Mine, vegetation rehabilitation area of the Heidaigou Coal Mine, and their corresponding control groups as the research targets. The results are shown in Figure 6.

For the mining subsidence area and control group of the Daliuta Coal Mine, the explanation degrees of RDA1 and RDA2 axes of soil bacterial communities for the results reached 26.63% and 10.62% at the level of phyla, respectively. However, all the sampling points of the mining subsidence area and the control group did not clearly separate into groups. Instead, they intertwine with each other. As per the RDA analysis results of soil physicochemical properties (Figure 6a), SWC positively correlated after the mining subsidence, while the sampling points of the control group positively correlated with ST, AP and AN. OM showed similar correlations in the mining subsidence area and the control group. The RDA analysis results of all phyla in samples (Figure 6b) indicate that the soil bacteria in the subsidence area were closely related to the phyla, including *Latescibacteria*, *Chloroflexi*, *Bacteroidetes*, *Proteobacteria*, and *Firmicutes*, while the soil bacteria in the control group were closely related to *Cyanobacteria*. Considering the phyla and physicochemical properties of sampling points (Figure 6c), pH and NN closely positively correlated with *Acidobacteria*, but correlated negatively with *Firmicutes* and *Verrucomicrobia* in a highly similar manner; OM correlated negatively with *Gemmatimonadetes*; EC correlated negatively with *Chloroflexi* and *Proteobacteria*; ST and AN correlated positively with *Armatimonadetes*; SWC correlated negatively with *Armatimonadetes*; and AK correlated positively with *Proteobacteria*.

The explanation degrees of RDA1 and RDA2 axes of soil bacterial communities for the vegetation rehabilitation and control group of the Heidaigou Coal Mine for the results reached 34.35% and 10.37% at the level of phylum, respectively. The sampling points of the vegetation rehabilitation area showed an obvious trend of clustering, while those of the control group were relatively scattered. According to the results of RDA analysis of soil physicochemical properties (Figure 6d), the vegetation rehabilitation area positively correlated with pH, SWC, OM, AP, AK, NN and AN, but was correlated negatively with EC and ST. In contrast, the sampling points of the control group had no evident relationship with these physicochemical properties. According to the RDA analysis of all the phyla and samples (Figure 6e), the soil bacteria in the vegetation rehabilitation area closely correlated with *Actinobacteria*, *Chloroflexi*, *Planctomycetes*, *Acidobacteria*, and *Nitrospira*, while the control group closely correlated with *Gemmatimonadetes*, *Proteobacteria*, and *Bacteroidetes*. Considering the phyla and physicochemical properties of the sampling points (Figure 6f), AK closely correlated positively with *Actinobacteria*, but negatively with *Gemmatimonadetes*; NN correlated closely positively with *Chloroflexi*, but negatively with *Bacteroidetes*; OM and AP closely positively correlated to *Planctomycetes* and *Acidobacteria*; ST closely positively related to *Proteobacteria* and *Bacteroidetes*; while EC closely positively correlated to other phyla except for *Actinobacteria,* which was negatively related.

These results thus show that the mining subsidence area of the Daliuta Coal Mine and the control group showed differences at the phylum level and in the physicochemical properties. The vegetation rehabilitation area and the control group of the Heidaigou Coal Mine exhibited similar characteristics. Mining subsidence and vegetation rehabilitation changed the distribution and physicochemical properties of the soil bacterial community structure.

#### 3.4.2. Interaction between OTUs and Physicochemical Properties in Mining Subsidence and Vegetation Rehabilitation Areas

The Mantel test results of OTU level of soil bacteria and environmental factors show that both ecological processes significantly changed the physical and chemical properties of soil, further affecting the diversity characteristics of soil bacterial communities (Table 3). However, the dominant environmental factors changing soil bacterial communities during different ecological processes varied. The OTU levels of soil bacteria had significantly positive correlations with NN in the subsidence area of the Daliuta Mine, but the OTU levels had no obvious correlation with other physicochemical properties, indicating that the loss of NN due to subsidence was an important driving force for the evolution of the bacterial community structure. There was a variation in dominant environmental factors for the soil bacteria in the vegetation rehabilitation area of Heidaigou Mine. The OTU levels of soil bacteria had significant correlations with ST, AP, AK, NN and AN. Although the nutrients in the soil (AP and NN) were still the dominant environmental factors for the development of bacterial communities, after vegetation rehabilitation, the coverage of plants on the surface increased, which significantly changed the ST and gas emissions. When the effect of environmental factors on the bacteria weakened, the cooperation between bacteria increased, resulting in a significant improvement in the stability of the soil environment.

#### 3.4.3. Interactions between Soil Bacterial Genera and Physicochemical Properties in the Mining Subsidence and Vegetation Rehabilitation Areas

**Figure 7:**
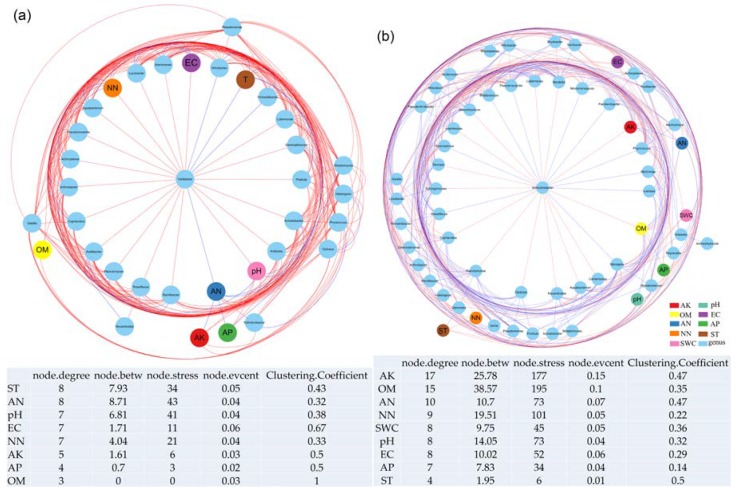
Radial layout plot between different genera and soil physicochemical properties in mining subsidence area (**a**) and vegetation rehabilitation area (**b**). node.degree: the topological property of a node in a network. node.betw: betweenness, the ratio of paths that pass through the node, which serves as a broker when it is high. node.stress: the number of geodesic paths that pass through one node. node.evcent: the degree of a central node that is connected to other central nodes. Clustering.Coefficient: hierarchical properties of networks, describing how well a node is connected with its neighbors. More detailed information can be found in Deng et al. [46].

For a further understanding of the interactions between soil bacterial species and environmental factors, the environmental factors were set as network nodes, and the top 50 genera of soil microorganisms in the mining subsidence area and the control group of the Daliuta Coal Mine were extracted to construct a network for the comparison of the connections between environmental factors and microorganisms, based on the RMT theory. The radial distribution results with *Variibacter* as the center of the network are shown in Figure 7a. The number of connections between nodes and strength of connections between ST and AN were the greatest and strongest, followed by those of pH, EC, and NN. The nodes of AN, pH and ST were also relatively strong. The connections between EC and ST nodes and other nodes were the closest. The connections between OM nodes and adjacent nodes were the strongest. The connections between soil physicochemical properties and soil bacterial species showed predominantly negative correlations in the interactions in this network. Additionally, as per Figure 7a, ST, pH, EC, NN and AN were directly and closely related to the selected strain *Variibacter*. *Variibacter* related negatively to pH, EC, and NN, but positively to ST and AN. Therefore, *Variibacter* could serve as the indicator strain for evaluating soil quality improvement after the subsidence in the Daliuta Coal Mine of the Loess Plateau.

Likewise, the environmental factors were set as network nodes, and the top 50 genera of soil bacteria in the vegetation rehabilitation area and the control group of the Heidaigou Coal Mine were extracted to construct a network based on the RMT theory for the comparison of the connections between environmental factors and microorganisms. The radial distribution results with *Solirubrobacter* as the center of the network are shown in Figure 7b. The number of connections between nodes and the strength of connections between AK and OM were the greatest and strongest, followed by those between AN, NN, SWC, pH, and EC. The connection between ST and bacterial communities were the least and the connections between soil physicochemical properties and soil microbial species showed primarily negative correlations between interactions in this network. In addition, OM and AK were directly and closely related to *Solirubrobacter*, showing negative correlations (Figure 7b). The remaining physicochemical properties were indirectly related to the strain. Hence, *Solirubrobacter* could serve as the indicator strain for evaluating soil quality improvement in the Heidaigou Coal Mine of the Loess Plateau.

## 4. Discussion

### 4.1. The Response of Ecological Processes to Mining Subsidence and Vegetation Rehabilitation in the Loess Plateau

Mining subsidence and vegetation rehabilitation are two important ecological processes in the entire life of coal mining and the most important factors influencing the local ecological environment [7,8,10,43]. The mining subsidence caused by coal mining has always been a global problem [7,56,57]. The changes in surface morphology increase the risks of soil and water loss, reduce the vegetation coverage and significantly interfere with the soil ecological environment [13,22,58,59,60,61]. Vegetation rehabilitation is an important measure of remedial engineering, that aims to increase vegetation coverage, reduce water and soil loss, improve the regional ecological environment, and enhance the public welfare of the mining area [62,63,64]. Vegetation rehabilitation significantly improved the primary soil physicochemical properties in the mining area, and further developed the soil bacterial communities [12,65,66]. Nearly half of the coal production capacity of China is concentrated in the Loess Plateau, but the local ecosystem has become very fragile. Damage due to mining usually worsens the local ecological environment, and vegetation rehabilitation effectively increases surface coverage, reduces soil erosion, and conserves soil fertility [13,22,58,59,60,61]. In this study, the structures, compositions, and interactions of soil bacterial communities in the mining subsidence area, vegetation rehabilitation area, and their corresponding control groups in the Loess Plateau were analyzed. These results will aid in the understanding the soil bacterial adaptability to the soil environments after mining subsidence and vegetation rehabilitation in the mining areas of the Loess Plateau. This work provides information on the screening of potential strains, with high adaptability for the remediation of the local ecosystems in the Loess Plateau.

### 4.2. Effects of Mining Subsidence and Vegetation Rehabilitation on the Soil Physicochemical Properties

Mining subsidence has a significantly complex impact on soil physicochemical properties. The changes in soil physicochemical properties in the mining subsidence area of the Daliuta Coal Mine show reduced OM, seriously depleted nutrients, and much declined soil fertility, consistent with the conclusions drawn in previous studies [22,23,26,67]. The clarification of these changes and their causes for mine ecological restoration is of great significance. After the mining subsidence, SWC and spatial variability increased. This is usually related to surface cracks and local ponding, which block the hydrological process at a sloped surface and reduce land productivity [68,69]. Further, ST and fluctuation decreased, which might be closely related to SWC [70,71]. However, pH increased slightly, which is related to the increase in SWC. The Ca^2+^ and Mg^2+^ ions in the soil are easier to precipitate and bond with OH-, thus increasing the alkalinity of soil [72,73].

Vegetation rehabilitation has a positive impact on soil physicochemical properties. The changes in soil physicochemical properties in the vegetation rehabilitation area of the Heidaigou Coal Mine indicate a slight increase in OM, and an increase in the contents of soil nitrogen, phosphorus, and potassium, consistent with the observations of previous studies [65,66]. Wang et al. [74] suggested that land reclamation could effectively improve the soil quality, and gradually improve the original level with the increase in reclamation period. In this study, soil physicochemical properties were also significantly positively affected by vegetation rehabilitation. SWC in the vegetation rehabilitation area was significantly higher than that in the control group, as per the results of Cao et al. [75]. Nevertheless, the improvement of SWC was assigned to human management in previous studies [4,9,10,22]. The decrease of ST in the vegetation rehabilitation area was attributed to loose soil, according to other studies [63,64,76,77]. Considering the experimental results of the present work, we prefer to correlate the decrease in ST with the decrease in bare soil, due to the increase in plant- coverage. The increase of soil pH in the vegetation rehabilitation area might be related to the materials used in field leveling or due to the improvement of soil moisture content to increase the contents of Ca^2+^ and Mg^2+^ ions in the soil solutions.

### 4.3. Changes in Soil Bacterial Communities after Mining Subsidence and Vegetation Rehabilitation

Mining subsidence was shown to exert a significant impact on soil bacterial diversity [78,79,80]. In the Daliuta Coal Mine, the abundance and homogeneity of soil bacterial communities decreased after mining subsidence, although there was no change in the primary bacterial phyla. Our results are consistent with those of de Quadros et al. [37] and Li et al. [81]. Mining subsidence changed the original support basement of soil environment, exhibiting a serious interference with the rhizosphere bacteria [78,79,82]. Soil bacteria were inevitably negatively affected [80]. However, due to technological limitations, the research on the interactions between soil bacteria subjected to mining subsidence has not been reported previously. In this study, the molecular ecological network analysis was adopted to explore the relationships between soil bacterial species. The results of this study show that the number of connecting lines, average clustering coefficient, average path distance and density of the soil bacterial molecular ecological network were higher than those of the control group, indicating enhanced complexity of the soil bacterial molecular ecological network after mining subsidence, the increased connectivity strength between nodes and the positive correlations, with closer connections between network nodes. These results indicate that soil bacteria can cooperate with each other to adapt to the nutrients loss in the unfavorable habitat caused by mining subsidence. However, the modularity index of the molecular ecological network of the mining subsidence area was smaller than that of the control group, indicating the weakened resistance of bacterial communities to external changes due to mining subsidence.

Vegetation rehabilitation can effectively improve the diversity of a soil bacterial community, as evidenced by this work and previous studies [29,39,40,42,82,83,84,85]. The number of connections between nodes after the reclamation was significantly smaller than that in the control group. Additionally, the number of network modules of the vegetation rehabilitation area was greater than that of the control group, and the relationship between modules changed from competition to cooperation. The network modularity index of the vegetation rehabilitation area was significantly higher than that of the control group, indicating significantly enhanced resistance of bacteria to external changes, and that the vegetation rehabilitation promoted the development of bacterial communities.

Furthermore, an analysis of the interactions between soil bacterial species after the mining subsidence and vegetation rehabilitation showed that after the variations or fluctuations of external environments, soil bacterial species cooperated with each other to adapt to the changing environments, although with similar ecological niches. This phenomenon plays an important role in the stable regulation and evolution of natural systems, and also reflects the sensitivity and adaptability of key species to the changes in the soil environment [86]. A similar trend was observed in the extreme environment threatened by CO_2_, and soil bacteria [49,50].

### 4.4. Interactions between Soil Bacterial Communities and Environmental Factors in both Ecological Processes

The soil bacteria were closely correlated to the physicochemical properties in both ecological processes. At the phylum level, the results of RDA analysis showed that the sampling points of the mining subsidence area and control group in the Daliuta Coal Mine intertwined without obvious differentiation. However, the sampling points of the vegetation rehabilitation area and control group in the Heidaigou Coal Mine differed spatially to a certain extent. The sampling points of the vegetation rehabilitation area showed obvious clustering, confirming the positive role of artificial restoration [85]. On the level of OTU, the soil bacterial communities in the mining subsidence area of the Daliuta Coal Mine correlated significantly positively with NN, indicating that the loss of soil nitrate-nitrogen caused by the subsidence might be the most important driving force for the evolution of the bacterial community structure. Soil bacterial communities of the vegetation rehabilitation area of the Heidaigou Coal Mine correlated significantly with ST, AP, AK, NN, and AN. Although soil nutrients (AP and NN) were still the dominant factors for the development of bacterial communities, the increase of plants coverage after reclamation significantly changed the soil temperature and gas emissions, weakened the impact of environmental factors on bacteria, enhanced the cooperation between bacterial communities, and significantly enhanced the stability of the soil environment.

The network constructed using genera and environmental factors showed that *Variibacter* in the Daliuta Coal Mine correlated negatively with pH, EC, and NN, but positively with ST and AN. This strain, therefore, has the potential for the monitoring and abatement of soil quality in subsidence areas, wherein, soil pH closely positively related to the dominant genus *Variibacter*. pH is a dominant factor for the difference in the horizontal structure of bacterial communities in the subsidence area of the Daliuta Coal Mine. This result is consistent with the conclusion drawn by Shen et al. [87], that the bacterial communities in the vertical zone of the Changbai Mountain were dominated by pH. Similar conclusions were also drawn by Dimitriu et al. [30], that after reclamation, the bacterial structures were mainly determined by pH and other soil abiotic characteristics. We also suggest that the difference in terrains caused by subsidence aggravated the differentiation of soil pH, water, and nutrients. This might further affect the development of soil bacterial communities. The molecular ecological network of the vegetation rehabilitation area in the Heidaigou Mine formed a radial distribution with a center of *Solirubrobacter*. OM and AK closely and negatively correlated with *Solirubrobacter*, showing that the nutrients, particularly OM and AK, were the dominant factors for the development of soil bacterial communities. Therefore, *Solirubrobacte* could serve as the dominant and crucial strain, indicating the improvement of the quality of sandy soil in the Loess Plateau.

### 4.5. Uncertainties of Soil Bacterial Development in Ecological Processes caused by Mining Activities

The effects of ecological processes due to mining activities on the development of soil bacterial communities still had several uncertainties. Geographical locations, meteorological conditions, seasons, mining intensity and other factors may cause the spatial heterogeneity in soil bacterial communities and physicochemical properties.

The geographic locations, or biogeography, can affect the development of soil bacterial communities [88,89,90,91,92,93,94]. Although both the Daliuta and Heidaigou Coal Mines are located at the northern edge of the Loess Plateau, they differed in soil bacterial diversity, community structure, and composition. In 1934, Bass-Becking reported that “Everything is everywhere, but the environment selects”, suggesting that bacteria have the characteristics of small size, large quantity, short generation cycle, high diffusion rate, and no restriction on diffusion [88,95]. Later, the distributions of soil bacteria were shown to be similar to those of large-size animals and plants [89,90], and these distributions were affected by regional environmental screening, interactions between species, and random diffusion screening [91,92,93,94]. These laws can also be applied to different areas disturbed by coal mining.

Meteorological conditions, especially temperature and precipitation, are important factors affecting bacterial diversity and community composition [96]. Temperature led to the differentiation of ammonia-oxidizing bacterial communities [97]. Low temperature also reduced the promotion effect of AM fungi on the nutrient uptake by plants [98]. The grasslands in North China are dry (AI < 0.32). With the increase of dryness index, the total number of nitrifying and denitrifying bacteria in soil increased, and the number of soil bacteria declined dramatically, due to seasonal drought [99]. Altitude also affects the composition of the bacterial community [100]. The meteorological conditions of the Daliuta and Heidaigou Coal Mines are different to a certain extent. Thus, the long-term meteorological conditions modified the soil bacteria, forming the stable states of soil bacterial communities.

Seasonal differences, i.e., the differences in the sampling times of soil bacteria, also interfered with the changes in soil bacterial community structures evaluated by sequencing. Previous studies have confirmed the effects of seasons on the diversity of soil bacterial communities [101,102,103]. Considering the interference of seasonal differences, the sampling in this study was performed from August 15-23, 2018, during which the soil bacteria were stably active.

Next, the mining intensity has a direct relationship with the mining subsidence, thus changing the physical and chemical properties of soil and further interfering with the soil bacterial communities [7,10,38,96,104].

The effects of several other possible factors on the soil bacterial communities were not negligible, and these effects are still under investigation. A quantitative study on the significance of these effects on soil bacterial species and their corresponding interactions is still required. Finally, the impact of some other possible factors affecting soil bacterial communities needs to be further recognized.

## 5. Conclusions

A comprehensive and systematic understanding of the evolution of soil bacterial communities in damaged mines is not only essential to the ecological restoration and abatement of the mining areas, but also crucial to improving the livelihood of the local residents and ecosystem services. In this study, field investigations, high-throughput sequencing, and molecular ecological network analyses were carried out to explore the changes in soil environment and differences in bacterial communities after the mining subsidence and reclamation in the Loess Plateau.

(1) Mining subsidence and vegetation rehabilitation exerted significant effects on soil physicochemical properties. Mining subsidence exhibited negative effects by decreasing the soil temperature and organic matter, available phosphorus, available potassium, and nitrate nitrogen. Vegetation rehabilitation exhibited positive effects by increasing the content of soil water, pH, EC, and contents of organic matter, available phosphorus, available potassium, nitrate-nitrogen, and ammonium nitrogen, but exerted a negative impact on soil temperature.

(2) The effects of mining subsidence and vegetation rehabilitation on the diversity of soil bacterial communities were rather varied. Mining subsidence led to a decrease of 20% in the Chao1 and ACE indexes of bacterial communities, while the vegetation rehabilitation led to a corresponding increase of 63% in these indexes. However, mining subsidence and vegetation rehabilitation did not change the dominant phyla in the soil, and the relative abundance of dominant phyla varied slightly.

(3) Mining subsidence and vegetation rehabilitation had a significant impact on the soil bacterial molecular ecological network. Mining subsidence increased the complexity of the connections within the network. The degree of concentration and number of connections of the network increased, with a concomitant increase in the positive correlations. While vegetation rehabilitation increased the diversification of the network, the connections were weakened.

(4) The interactions between soil bacteria changed to adapt to the interference due to mining. Mining subsidence promoted the cooperation between bacterial genera for the adaption to survival. Vegetation rehabilitation promoted the complexity of modules and the cooperation between bacteria to acquire more external resources. *Variibacter* could serve as the indicator strain for soil quality improvement after the subsidence, and *Solirubrobacte* could be adopted as an indicator to assess the improvement of the quality of sandy soil, but this hypothesis needs further study.

## Figures and Tables

**Table 1:** Changes of soil physicochemical properties after mining substance and vegetation rehabilitation.

Soil Physicochemical Properties	Mining Substance		Vegetation Rehabilitation	
	LS	CLS	LR	CLR
SWC/(%)	10.01 ± 3.21*	7.52 ± 1.74	13.35 ± 3.64*	10.67 ± 2.00
ST/(°C)	26.94 ± 1.65**	31.00 ± 4.37	26.15 ± 3.54***	31.32 ± 3.62
pH	7.71 ± 0.44	7.48 ± 0.42	8.56 ± 0.27***	7.74 ± 0.45
EC/(ms·cm^−3^)	5.76 ± 1.12	5.41 ± 1.81	15.87 ± 3.49*	12.35 ± 3.43
OM/(g·kg^−1^)	0.95 ± 0.63**	1.65 ± 0.51	1.86 ± 0.45**	1.46 ± 0.23
AP/(mg·kg^−1^)	123.14 ± 22.43*	145.14 ± 22.66	130.48 ± 31.53*	96.13 ± 38.13
AK/(mg·kg^−1^)	99.26 ± 8.38***	108.89 ± 4.21	74.94 ± 15.74**	51.25 ± 17.07
NN/(mg·kg^−1^)	0.29 ± 0.11	0.31 ± 0.13	0.51 ± 0.24**	0.29 ± 0.06
AN/(mg·kg^−1^)	0.42 ± 0.17*	0.60 ± 0.19	1.22 ± 0.18*	0.98 ± 0.38

Note: * represents the significance of the disturbance zone and the control T-test, * mean significant difference at 5%, ** mean significant difference at 1%, *** mean extremely significant difference at 1%. SWC, soil water content; ST, soil temperature; OM, soil organic matter; AP, available phosphorus; AK, available potassium; NN, nitrate-nitrogen; AN, ammonium nitrogen. The abbreviations in the text have the same meaning.

**Table 2:** Topological properties of soil bacterial molecular ecological networks in mining subsidence and vegetation rehabilitation.

Topological Parameters	Mining Subsidence		Vegetation Rehabilitation	
	LS	CLS	LS	CLS
Similarity threshold ^1^	0.81	0.81	0.84	0.84
Nodes ^2^	245	171	114	92
Links ^3^	514	162	148	320
AvgK ^4^	4.196	1.895	2.596	6.957
AvgCC ^5^	0.244	0.168	0.213	0.414
AvgPD ^6^	6.007	5.333	6.241	4.983
Density ^7^	0.017	0.011	0.023	0.076
Connectivity ^8^	0.647	0.087	0.295	0.957
Module ^9^	29	40	22	4
Modularity ^10^	0.652	0.915	0.777	0.483
R square ^11^	0.921	0.888	0.939	0.700

Note: ^1^ Similarity threshold: the soil bacterial molecular ecological network of the experimental group and the control group were built under the same threshold when *P < 0.05*. ^2^ Nodes: the numbers formed by the network construction. ^3^ Links: the number of connections between nodes. ^4^ AvgK: Average connectivity, which presented the complexity of networks.^5^ AvgCC: Average clustering coefficient, which was used to determine the extent of the module structure present in different networks. ^6^ AvgPD: Average path distance, or the average distance between two nodes. ^7^ Density: the complexity of the network. ^8^ Connectivity: node degree, was used to describe the topological property of a node in a network. ^9^ Module: a group of OTUs that had high connections among themselves, but fewer connections outside the group in the network. ^10^ Modularity: a network that could be naturally divided into communities or modules. ^11^ R square: the credibility of scale-free networks. More information about these parameters is given in Deng et al. [46].

**Table 3:** Mantel Test of the soil bacterial community, in terms of OTU level and environmental factors.

Soil Physicochemical Properties	Mining Substance		Vegetation Rehabilitation	
	LS	CLS	LR	CLR
SWC/(%)	−0.0075	0.0197	−0.00365	−0.0282
ST/(°C)	−0.0253	0.0059	−0.0227	0.0875*
pH	−0.0229	−0.019	0.0309	0.0023
EC/(ms·cm^−3^)	−0.0083	−0.0727	0.02148	−0.0257
OM/(g·kg^−1^)	−0.0692	−0.0651	−0.05059	0.0376
AP/(mg·kg^−1^)	−0.0671	−0.0153	0.175**	0.0582
AK/(mg·kg^−1^)	−0.0092	0.0116	−0.0133	0.0823*
NN/(mg·kg^−1^)	0.1032*	−0.0707	0.1543**	−0.033
AN/(mg·kg^−1^)	−0.0329	0.0041	−0.004332	0.122*

Note: * mean significant difference at 5%, ** mean significant difference at 1%. All significant points are highlighted in bold.
